# The Pharmaco –, Population and Evolutionary Dynamics of Multi-drug Therapy: Experiments with *S. aureus* and *E. coli* and Computer Simulations

**DOI:** 10.1371/journal.ppat.1003300

**Published:** 2013-04-04

**Authors:** Peter Ankomah, Paul J. T. Johnson, Bruce R. Levin

**Affiliations:** 1 Molecules to Mankind Program and Graduate Program in Population Biology, Ecology and Evolution, Laney Graduate School, Emory University, Atlanta, Georgia, United States of America; 2 Department of Biology, Emory University, Atlanta, Georgia, United States of America; Tufts University School of Medicine, United States of America

## Abstract

There are both pharmacodynamic and evolutionary reasons to use multiple rather than single antibiotics to treat bacterial infections; in combination antibiotics can be more effective in killing target bacteria as well as in preventing the emergence of resistance. Nevertheless, with few exceptions like tuberculosis, combination therapy is rarely used for bacterial infections. One reason for this is a relative dearth of the pharmaco-, population- and evolutionary dynamic information needed for the rational design of multi-drug treatment protocols. Here, we use *in vitro* pharmacodynamic experiments, mathematical models and computer simulations to explore the relative efficacies of different two-drug regimens in clearing bacterial infections and the conditions under which multi-drug therapy will prevent the ascent of resistance. We estimate the parameters and explore the fit of Hill functions to compare the pharmacodynamics of antibiotics of four different classes individually and in pairs during cidal experiments with pathogenic strains of *Staphylococcus aureus* and *Escherichia coli*. We also consider the relative efficacy of these antibiotics and antibiotic pairs in reducing the level of phenotypically resistant but genetically susceptible, persister, subpopulations. Our results provide compelling support for the proposition that the nature and form of the interactions between drugs of different classes, synergy, antagonism, suppression and additivity, has to be determined empirically and cannot be inferred from what is known about the pharmacodynamics or mode of action of these drugs individually. Monte Carlo simulations of within-host treatment incorporating these pharmacodynamic results and clinically relevant refuge subpopulations of bacteria indicate that: (i) the form of drug-drug interactions can profoundly affect the rate at which infections are cleared, (ii) two-drug therapy can prevent treatment failure even when bacteria resistant to single drugs are present at the onset of therapy, and (iii) this evolutionary virtue of two-drug therapy is manifest even when the antibiotics suppress each other's activity.

## Author Summary

In this study, we combine pharmacodynamic experiments using pathogenic strains of *E. coli* and *S. aureus* with mathematical and computer simulation models to explore the relative efficacies of two-drug antibiotic combinations in clearing infections and preventing the emergence of resistance. We develop a pharmacodynamic method that provides a convenient way to determine whether drug combinations will interact synergistically, antagonistically, additively or suppressively. We find that it is not possible to predict the nature and form of drug interactions based on what is known about the mode of action of individual drugs, thus illustrating the necessity of assessing the efficacy of drug combinations empirically. Our simulations of the within-host population and evolutionary dynamics of bacteria undergoing multi-drug treatment indicate that the form of the interaction between drugs observed experimentally can substantially affect the rate of clearance of the infection. On the other hand, the form of these interactions plays a minimal role in the emergence of resistance. Even when antibiotics are suppressive, two-drug therapy can prevent the ascent of bacteria resistant to single drugs that are present at the start of therapy and/or generated during the course of the infection.

## Introduction

The simultaneous use of multiple anti-microbial agents is standard for the treatment of long-term infectious diseases like tuberculosis and HIV/AIDS [1], [2]. Multiple drugs are also used to treat polymicrobial infections and in situations where the etiologic agent of an infection is unknown at the start of therapy [3]. Increasingly, this “combination therapy” is being used for the treatment of other chronic bacterial infections like endocarditis, osteoarticular infections and osteomyelitis as well as sepsis [4]–[6].

The motivation for treating with multiple, rather than single drugs, has both evolutionary and pharmacological components. Theoretically, if multiple drugs with different modes of action are used for treatment, bacteria resistant to each single drug, if present, will remain susceptible to the other drugs. Hence, multi-drug therapy would be less likely to be thwarted by the evolution of resistance than monotherapy. This intuitively appealing evolutionary reason for combination therapy is supported by evidence [7]–[14] as well as logic. From a pharmacodynamics (PD) perspective, there are at least two potential virtues for combination therapy. The drugs can be synergistic in their action and provide greater cidal activity than single drugs at comparable doses. Combining drugs can also result in increased antimicrobial activity without elevating single-drug concentrations to levels that engender debilitating side effects. In some situations, the *in vitro* synergy of multiple treating drugs is positively correlated with bactericidal activity and clinical outcome [15]–[20] and, at the same time, antagonistic interactions between drugs *in vitro* can negatively impact therapeutic success [21]–[23].

As appealing as the reasons for multi- rather than single drug therapy may be, the clinical utility of combination therapy remains equivocal for many infections [24]. One of the reasons for this is the relative dearth of sufficient answers to a number of fundamental questions. How does one know whether a specific combination therapy regimen will be more or less effective than monotherapy for a specific infection? How does one quantify the pharmacodynamics of multiple drugs? Are there generalizable rules about how drugs of different classes interact? Under what conditions will the collective activity of multiple drugs exceed their individual activity? How do the pharmacological interactions between drugs in combination affect the emergence of resistance during the course of therapy?

Although these questions have been addressed in various ways, at this juncture the answers obtained are restrictive. Checkerboard titrations and time kill assays seem to be the most popular *in vitro* methods to evaluate the form of interactions between antibiotics (synergy, antagonism, suppression or additivity). The checkerboard assay generates a single parameter, the Fractional Inhibitory Concentration (FIC) index as a measure of the efficacy of drug combinations relative to their respective individual efficacies measured by the Minimum Inhibitory Concentration, MIC [25]. Time-kill assays express the efficacy of drug combinations in terms of the log-fold reduction in viable cell density generated by these combinations relative to the most active single agent over an arbitrary time period [26]. Neither of these measures of the combined action of drugs provides information about the functional relationship between the concentrations of these drugs and the rate at which the target bacteria are killed [27]. The dynamics of antibiotic-mediated killing by pairs of drugs with the same FIC index and/or log-fold reductions in viable cells can differ profoundly and these single parameter measures may not provide an adequate picture of the cidal properties of drug combinations for the design of antibiotic treatment regimens. Another limitation of this single interaction parameter approach is that it fails to account for the changes in the form of the interaction with changing concentrations of the drug, pharmacokinetics [28]–[31].

The relationship between the concentration of single bactericidal antibiotics and the rate of growth or death of bacteria during the initial exposure can be fit to Hill functions [27], [31], but at this juncture it is unclear how these or other pharmacodynamic functions can account for the complication of the interactions between drugs. To our knowledge, there is no *a priori* way to quantitatively predict how multiple drugs will interact from their single drug pharmacodynamics. Although there have been some compelling analyses of the pharmacodynamics of multiple antibiotics and bacteria, with few exceptions e.g. [31], [32] these have been restricted to low and often sub-MIC and thereby sub-therapeutic concentrations of these drugs [33], [34].

Finally, there is the phenomenon of persistence. Antibiotic-mediated killing is a biphasic process: the rate of bactericidal activity during *in vitro* time-kill experiments declines with time and approaches zero. Depending on the drug employed, a substantial fraction of genetically susceptible but phenotypically resistant bacteria, the persisters, survive [35], [36]. A comprehensive consideration of the pharmacodynamics of combination therapy would also provide information about how multiple drugs affect the level of persistence. Bar two recent exceptions [37], [38], all studies of persistence of which we are aware have focused solely on single drugs.

In this report we develop, illustrate and evaluate a procedure that addresses these quantitative questions of the pharmaco- and evolutionary dynamics of multi-drug antibiotic therapy. Using *in vitro* experiments with *Staphylococcus aureus* and *Escherichia coli*, we determine the functional relationship between the concentrations of four antibiotics of different classes (singly and in pairs) and the rate of growth/kill of these bacteria during the exponential phase of their confrontations with these drugs. Using this method, we are able to explore the pharmacodynamics of multiple drugs at supra- as well as sub-MIC concentrations. We also evaluate the relationship between cidal concentrations of these antibiotic combinations and the density of persisters surviving exposure to the drugs. To explore the potential clinical implications of the experimental PD results, we employ a mathematical model of multi-drug therapy that allows for the evolution of resistance to the treating drugs. Using computer simulations with parameter values in the ranges of those estimated from the experimental analyses, we explore the effects of two-drug PD efficacy on the rate of clearance of infections and the emergence of single- and multi-drug resistance.

## Results

### Multi-drug pharmacodynamics in theory

We open this section with an *a priori* consideration of the pharmacodynamics of two drugs for qualitatively different forms of interactions between these drugs. As our measure of the concentrations for pairs of drugs, in theory and in practice, we use a single variable×CU (×multiples of “Cidal Units”), which is calculated as the sum of equal multiples of the MICs of each single drug. For example, if the MIC of drug A is 1 µg/mL and that of B 2 µg/mL, for the pair, 2×CU is the combination of 1 µg/mL of A and 2.0 µg/mL B. Implicit in this measure is a null assumption of Loewe's additivity [39] which assumes that the magnitude of the killing effect of additive multiple drugs is proportional to that which would result from the sum of equipotent concentrations of each drug separately. For instance, under this assumption, the combination of 0.5×MIC each of two additive drugs, ×CU = 1, would be equal to 1×MIC of each of the antibiotics on their own [40].

**Figure 1:**
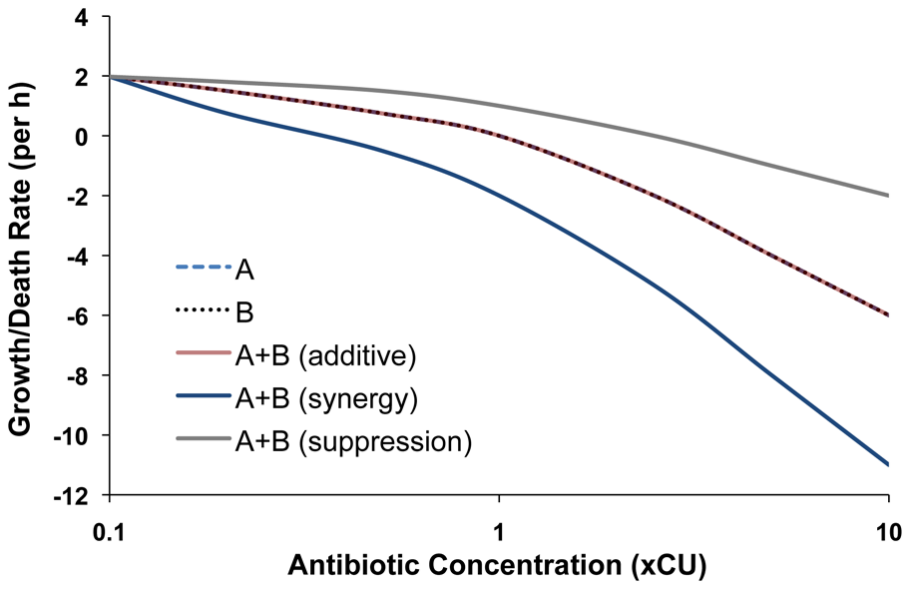
Anticipated single and two-drug Hill functions for qualitatively different types of drug interactions. Hill functions of single antibiotics (A or B) and combinations (A+B) representing synergy, additivity and suppression are shown. The growth and death rates used for these illustrations are in the range of those observed experimentally.

Using the xCU's as measures of the concentrations of single and pairs of drugs and a method similar to that used in Regoes *et al.* [27] (See Materials and Methods), it is possible to fit Hill functions to the rate of bacterial killing during the exponential phase of kill. In Figure 1, we illustrate the form of the Hill functions that would be anticipated for single drugs (A or B) and qualitatively different types of two drug interactions (A+B). In this idealized case, if (i) the drugs are additive at each concentration, the rate of kill generated by the two drugs together is identical to that of each of the drugs alone; (ii) the drugs are suppressive, their combined rate of kill is less than that of each of the single drugs alone, and (iii) the drugs are synergistic, their combined rate of kill is greater than that for the individual drugs. It should be noted that in this illustration, per our assumption of Loewe additivity, the single drug Hill functions are identical and the same as that for a purely additive drug combination. In generating Figure 1, we assumed a directly proportional relationship between antibiotic concentration and the rate of kill engendered. In theory, more complex relationships between drug concentration and rate of antibiotic-mediated killing can occur, and as seen from the below experimental results, do obtain.

### Multi-drug pharmacodynamics in practice

We performed time-kill experiments using single and two-drug combinations to determine the relationship between the concentrations of these drugs and the rate of kill of the target bacteria (Figures S1, S2, S3, S4). Ampicillin, ciprofloxacin, tobramycin and tetracycline were used in the *E. coli* experiments and oxacillin, vancomycin, ciprofloxacin and gentamicin in experiments with *S. aureus*. For both single and multiple drugs, we observed biphasic cidal dynamics; an exponential decline in bacterial survival followed by a leveling off period with minimal cidal activity.

We fit Hill functions to the concentration-dependent rate of kill of bacteria during the exponential phase of killing in our experiments, between 0 and 1 hour for *E. coli* and between 0 and 4 hours for *S. aureus*. We estimated the Hill function parameters for each of the four single antibiotics and six pairs of antibiotics used in the time-kill experiments with both bacteria. As the equivalent of the pharmacodynamic, Hill function estimate of the MIC for single drugs, we determined the analogous Hill function estimate for pairs of drugs, which we call the realized MIC, rMIC. We list our estimates of these parameters in Tables S1 and S2.

**Figure 2:**
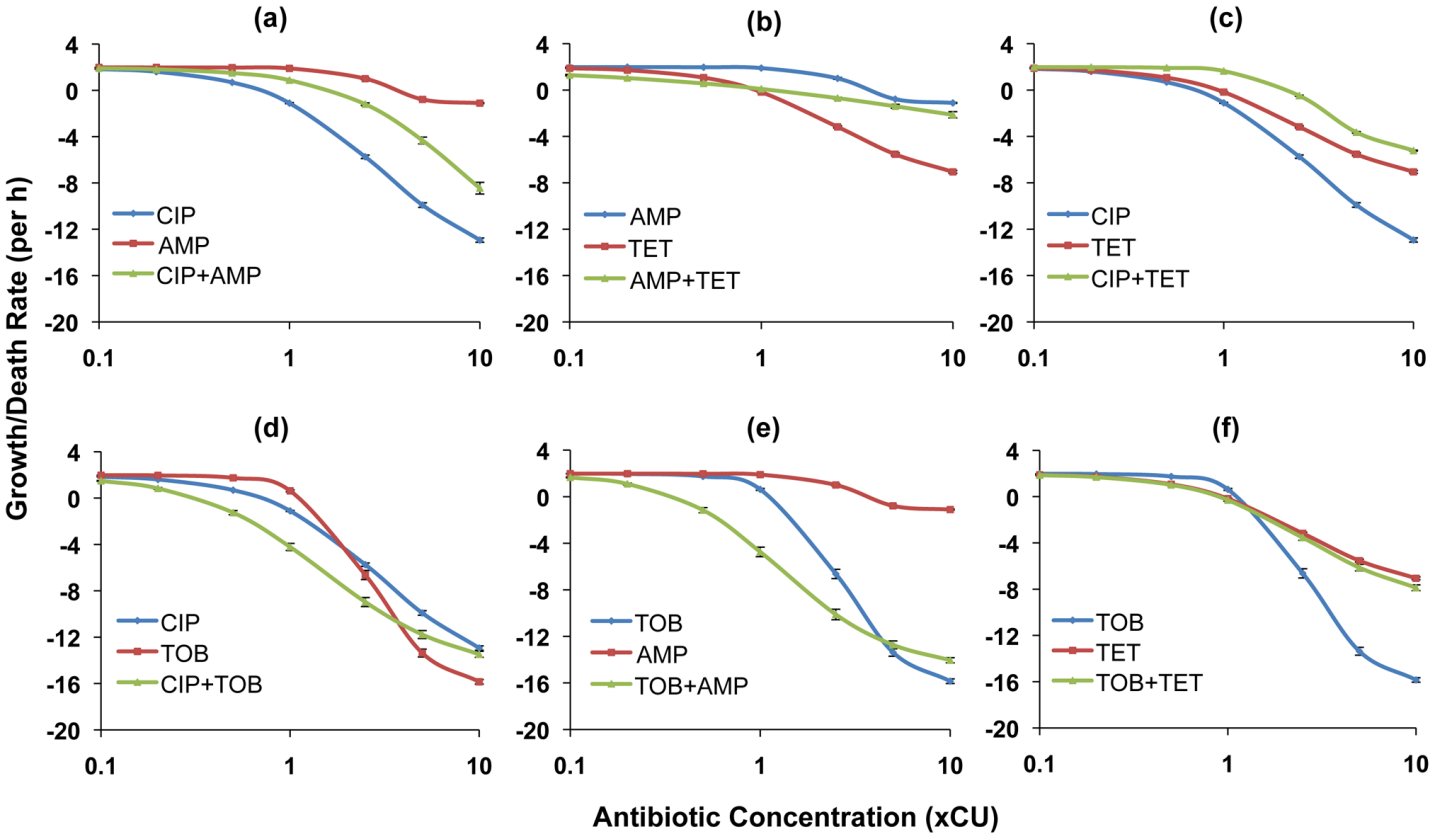
Hill functions for two-drug combinations and the constituent individual antibiotics (*E. coli*). Each graph shows the Hill functions for a drug combination and the constituent single drugs with drug concentrations normalized as multiples of Cidal Units (xCU). Error bars represent the standard errors for the growth/death rate at each antibiotic concentration. (a) ampicillin (AMP), ciprofloxacin (CIP), and ampicillin+ciprofloxacin (b) ampicillin, tetracycline (TET), and ampicillin+tetracycline (c) ciprofloxacin, tetracycline, and ciprofloxacin+tetracycline (d) ciprofloxacin, tobramycin (TOB), and ciprofloxacin+tobramycin (e) tobramycin, ampicillin, and tobramycin+ampicillin (f) tobramycin, tetracycline, and tobramycin+tetracycline.

**Figure 3:**
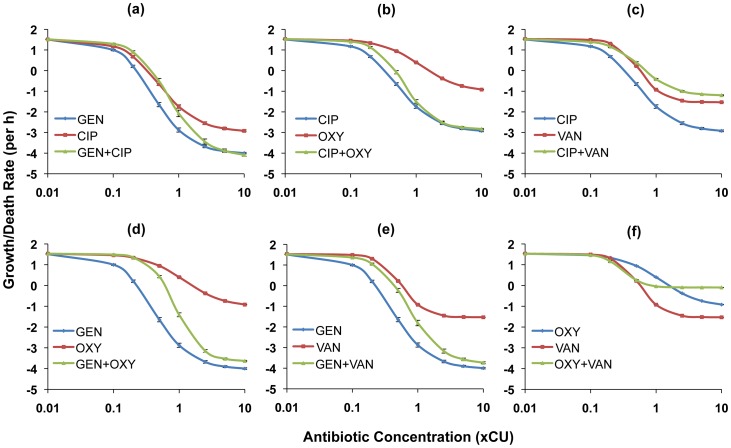
Hill functions for two-drug combinations and the constituent individual antibiotics (*S. aureus*). Each graph shows the Hill functions for a drug combination and the constituent single drugs with drug concentrations normalized as multiples of Cidal Units (xCU). Error bars represent the standard errors for the growth/death rate at each antibiotic concentration. (a) ciprofloxacin (CIP), gentamicin (GEN), and ciprofloxacin+gentamicin (b) ciprofloxacin, oxacillin (OXY), and ciprofloxacin+oxacillin (c) ciprofloxacin, vancomycin (VAN), and ciprofloxacin+vancomycin (d) gentamicin, oxacillin, and gentamicin+oxacillin (e) gentamicin, vancomycin, and gentamicin+vancomycin (f) oxacillin, vancomycin, and oxacillin+vancomycin.

In Figures 2 and 3, we show the PD functions for all two-drug combinations together with the corresponding single-drug PDs for the component antibiotics. For *E. coli* there was no detectable cidal activity at antibiotic concentrations less than 0.1×CU and we use 0.1×CU as the minimum concentration (Figure 2). Since we observed cidal activity at lower drug concentrations for *S. aureus* (a consequence of lower rMIC's), we use 0.01×CU as the minimum concentration (Figure 3).

For *E. coli*, combining ampicillin with any drug yielded a greater rate of kill than ampicillin alone at comparable concentrations. The ampicillin+ciprofloxacin (Figure 2a) and ampicillin+tetracycline (Figure 2b) combinations were generally intermediate in efficacy between the component single antibiotics (a qualitative result we designate as antagonism), while the ampicillin+tobramycin combination (Figure 2e) exhibited synergy at most concentrations. When used in combination, tetracycline diminished the cidal activity of the two most efficacious antibiotics, ciprofloxacin and tobramycin. In combination with ciprofloxacin a suppressive interaction prevailed (Figure 2c), while for the tobramycin+tetracycline combination, the two drugs together exhibited the same efficacy as tetracycline alone (Figure 2f). The combination of tobramycin with ciprofloxacin exhibited synergistic interactions at concentrations below approximately 5×CU. At greater concentrations than this, the single antibiotic tobramycin was more effective than when used in combination (Figure 2d).

For *S. aureus*, most antibiotic combinations were either intermediate in efficacy between the individual drugs or generated cidal activity equivalent to that of the more effective of the constituent drugs (Figures 3a,b,d,e). We observed suppressive interactions at higher concentrations when vancomycin was combined with either ciprofloxacin (Figure 3c) or oxacillin (Figure 3f). Indeed, for the latter combination, the two individually bactericidal drugs became bacteriostatic. It is also worth noting that save for the representative beta-lactams, the maximal death rates exhibited in the *S. aureus* experiments for all drugs/drug pairings were substantially lower than those observed in the *E. coli* experiments.

### Persistence

**Figure 4:**
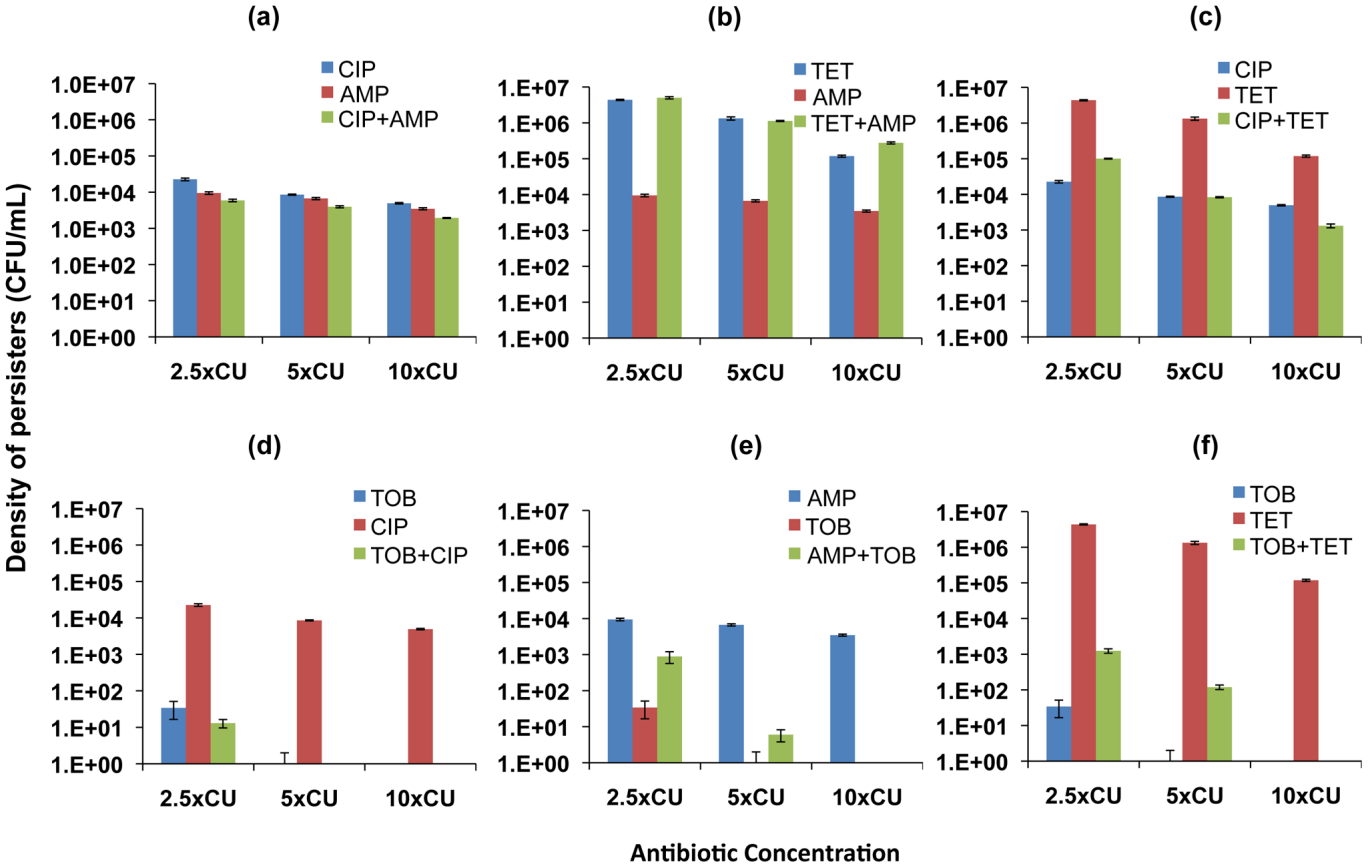
Density of persisters for two-drug combinations and the constituent individual antibiotics (*E. coli*). Viable cell densities of *E. coli* following 6 hours of exposure to equivalent cidal concentrations of single drugs and two-drug combinations (mean and standard error for 10 independent cultures shown). (a) ampicillin (AMP), ciprofloxacin (CIP), and ampicillin+ciprofloxacin (b) ampicillin, tetracycline (TET), and ampicillin+tetracycline (c) ciprofloxacin, tetracycline, and ciprofloxacin+tetracycline (d) ciprofloxacin, tobramycin (TOB), and ciprofloxacin+tobramycin (e) tobramycin, ampicillin, and tobramycin+ampicillin (f) tobramycin, tetracycline, and tobramycin+tetracycline.

**Figure 5:**
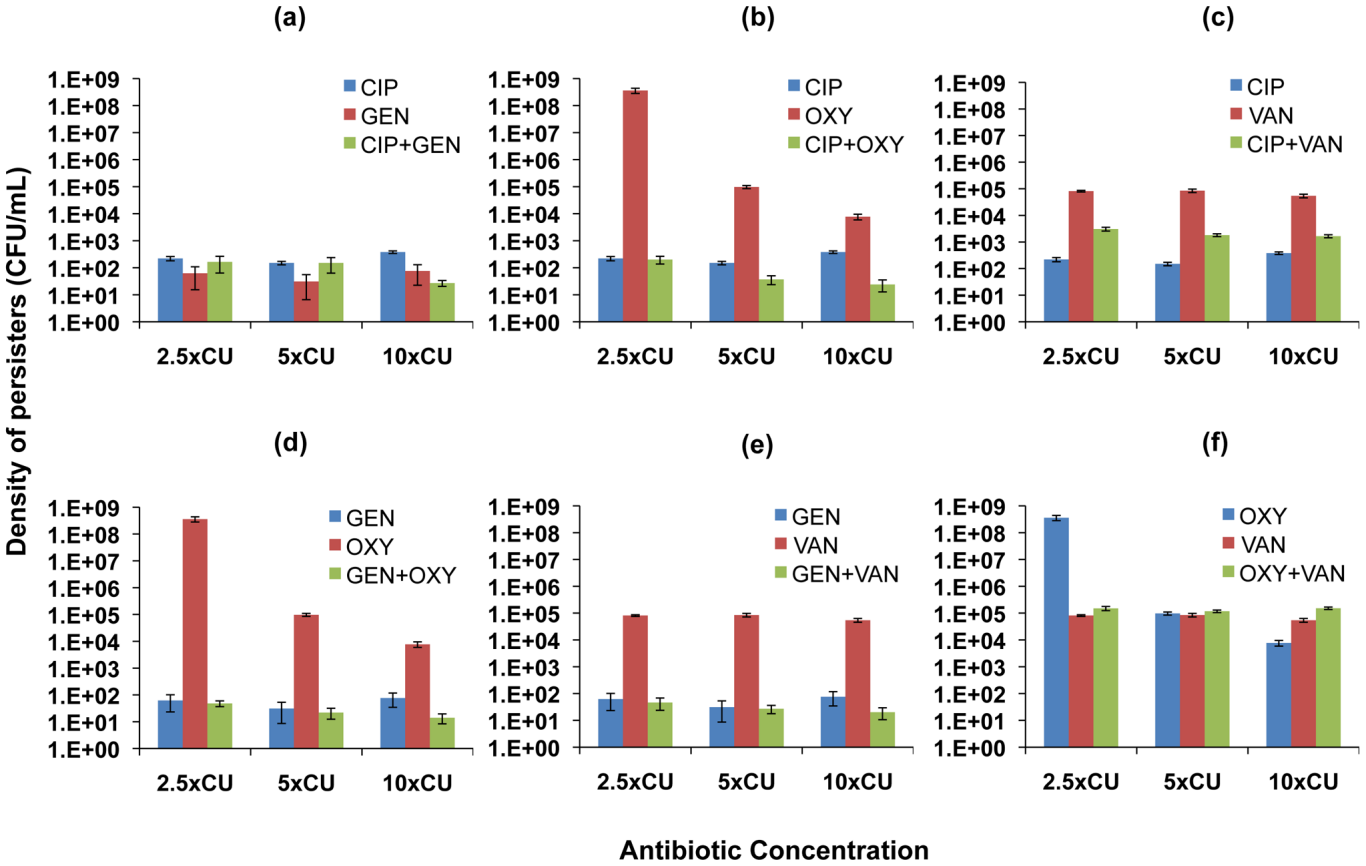
Density of persisters for two-drug combinations and the constituent individual antibiotics (*S. aureus*). Viable cell densities of *S. aureus* following 22 hours of exposure to equivalent cidal concentrations of single drugs and two-drug combinations (mean and standard error for 10 independent cultures shown). (a) ciprofloxacin (CIP), gentamicin (GEN), and ciprofloxacin+gentamicin (b) ciprofloxacin, oxacillin (OXY), and ciprofloxacin+oxacillin (c) ciprofloxacin, vancomycin (VAN), and ciprofloxacin+vancomycin (d) gentamicin, oxacillin, and gentamicin+oxacillin (e) gentamicin, vancomycin, and gentamicin+vancomycin (f) oxacillin, vancomycin, and oxacillin+vancomycin.

Hill functions provide good fits for the initial exponential phase of time-kill curves but not for the second phase during which the rate of killing declines and the viable cell population is dominated by persisters. In an effort to examine how two-drug therapy affects levels of persisters, we extended our analysis to the relationship between single and two-drug treatment regimens and the density of persisters present after exposure to the drugs. In Figures 4 and 5, we show persistence levels for drug combinations and the component single antibiotics of each combination. The average CFU's and standard errors for ten independent replicate cultures of 2.5×, 5× and 10×CU treatments sampled at 6 h for *E. coli* (Figure 4) and 22 h for *S. aureus* (Figure 5) are shown.

For *E. coli*, similar densities of persisters were observed for ciprofloxacin and ampicillin used individually as well as in combination (Figure 4a). Tetracycline used on its own resulted in the highest level of persistence among all the antibiotics studied. When combined with ampicillin, the density of persisters observed was similar to that generated by tetracycline alone. This result occurred despite the observation that treating with the other antibiotic in the combination, ampicillin, led to a lower level of persistence compared to tetracycline (Figure 4b). Combining ciprofloxacin and tetracycline, however, led to lower levels of persistence than equivalent concentrations of tetracycline (Figure 4c). Among all the antibiotics, tobramycin was the most effective in reducing the level of persisters. We recovered persisters only at 2.5×CU in treatments with tobramycin. When combined with ciprofloxacin, the combination was more effective than ciprofloxacin used singly and just as effective as tobramycin alone (Figure 4d). Combining tobramycin with ampicillin (Figure 4e) and tetracycline (Figure 4f), on the other hand, decreased the efficacy of tobramycin.

In the *S. aureus* experiments, gentamicin and ciprofloxacin used singly resulted in lower levels of persistence than oxacillin and vancomycin (Figures 5a,f). Strikingly, cultures exposed to the presumptively cidal 2.5×CU of oxacillin had, by 22 hours, grown to the same densities as antibiotic-free controls (Figure 5b). This result can be attributed to a decline in the effective concentration of this drug, rather than mutations to resistance [41]. However, combinations of 1.25×MIC of oxacillin with 1.25×MIC of any of the other drugs exerted a cidal effect, and the cultures did not grow (Figures 5b,d,f). When gentamicin was present in the drug pair, for all combinations of two drugs the level of persistence was at least as low as when gentamicin was used alone (Figures 5a,d,e). Combinations involving ciprofloxacin generated densities of persisters either equivalent to that generated by ciprofloxacin alone (Figures 5a,b) or intermediate between those generated by the individual antibiotics (Figure 5c).

### Potential clinical implications

What are the implications of the preceding pharmacodynamic results for the design and evaluation of antibiotic treatment regimens and the emergence of antibiotic resistance? To begin to address these questions we use a simple mathematical model of the within-host pharmacokinetics, population and evolutionary dynamics of bacteria undergoing multi-drug therapy.

### The model

The model used here is a variant of that used in [42]. It considers two antibiotics with concentrations and designations, A and B, and two subpopulations of bacteria; one that is actively replicating and one that is not (the persisters), with densities and designations, S and P, respectively. Bacteria can be of one of four different genotypic resistance profiles: they can be susceptible to the action of both antibiotics, susceptible only to A or B and resistant to the other, or resistant to both. Note though, that any bacterium in a persister state exhibits a phenotypic refractoriness to antibiotic action regardless of its genotypic resistance profile.

Persisters are generated from S cells in a stochastic manner which we simulate via the following Monte Carlo procedure: the maximal rate of persister production is set at *f* per cell per hr, and if f*S*Δt is greater than the value of a rectangularly-distributed random number between 0 and 1, then one individual is lost from the S population and one gained by the P population. The step size of an Euler simulation, Δt, is chosen so that the probability of generating a persister is less than 1. The transition from persisters back to growing cells is simulated in a similar fashion, with a maximal rate of *g* per cell per hour, where *g<f*. Single- and two-drug resistant bacteria are also generated via a similar Monte Carlo procedure, with maximal rates of mutant production *μ_A_* and *μ_B_*, representing mutation rates to resistance for antibiotics A and B respectively.

We represent the pharmacodynamics of both single and combined antibiotic action (i.e. treating with Antibiotic A, B, or both) with a Hill function, as per the preceding experimental analyses. For pharmacokinetics, we assume regular antibiotic input of A_max_ and B_max_ µg/mL every *T* hours. The effective concentration of these drugs decline at rates *d_A_* and *d_B_* µg/mL per hour. Net bacterial growth depends on the efficacy of antibiotic cidal action as well as on the availability of a limiting resource of concentration *R* µg/mL. We assume a continuous flow of this resource from a reservoir where it is maintained at a concentration *C* µg/mL. This resource enters the host at a rate *w* per mL per hour, which is the same rate at which antibiotics, bacteria, resources and wastes are washed out. The rate of bacterial replication is a monotonically increasing function of *R* with a half-saturation coefficient of *k_m_* µg/mL [43]. Conversion of resources into bacterial cells occurs at a conversion efficiency of *e* µg/cell. For the numerical analysis of the properties of this model, computer simulations, we use Berkeley Madonna. Copies of the program can be obtained from www.eclf.net/programs.

The standard values and/or ranges of the parameters and variables considered in our numerical analysis of the properties of this model are presented in Table 1. We note here that this simple mathematical model is not intended as a quantitatively precise analogue of a specific disease and treatment process but rather to provide a schema for assessing the potential clinical implications of our *in vitro* pharmacodynamic results. Whenever possible, the parameter values used are in the range of those estimated from the experimental analyses. Parameters not specific to this study are within the range of those used in other pharmacodynamic and pharmacokinetic studies of antimicrobial therapy [27], [42], [44].

**Table 1:** Values and ranges for variables and parameters used for generating numerical solutions.

Variable/Parameter	Description	Value or range considered*
Variables		
A, B	Antibiotic concentration (µg/mL)	0–10
S_X_	Density of planktonic bacteria sensitive to both antibiotics, x = 0; resistant to A, x = RA; resistant to B, x = RB; and resistant to A and B, x = RAB (cells per mL)	1–10^10^
P_X_	Density of persisters sensitive to both antibiotics, x = 0; resistant to A, x = RA; resistant to B, x = RB; and resistant to A and B, x = RAB (cells per mL)	1–10^10^
*R*	Concentration of the limiting resource (µg/mL)	0–1000
Parameters		
*ψ_max_*	Maximum hourly growth rate of replicating bacteria	(1.5)
*ψ_miny_*	Maximum hourly death rate of antibiotic y, where y = A, B and AB (A+B)	−1–−15
*MIC_y_*	Minimum Inhibitory Concentration of antibiotic y, where y = A, B and AB (A+B) (µg/mL)	(1)
*κ_y_*	Hill coefficient of antibiotic y, where y = A, B and AB (A+B)	(1)
*w*	Hourly washout rate	(0.2)
*f*	Hourly rate at which S is converted into P	10^−2^ or 10^−5^
*g*	Hourly rate at which P is converted into S	10^−3^ or 10^−6^
*C*	Reservoir resource concentration (µg/mL)	(1000)
*e*	Efficiency of resource conversion into cells (µg/cell)	(5×10^−7^)
*k_m_*	Concentration of resource at half maximal growth (µg/mL)	(0.25)
A_max_, B_max_	Antibiotic concentration added at each dosing period (µg/mL)	(5)
*d_A_*, *d_b_*	Antibiotic decay rate (h^−1^)	(0.1)
*T*	Time between doses (h)	(12)
*μ_A_*, *μ_B_*	Mutation rate (mutations per cell division)	10^−8^

* Values in parentheses are the standard values used for numerical analysis of the model.

### Single and multi-drug therapy and the contribution of persistence levels

**Figure 6:**
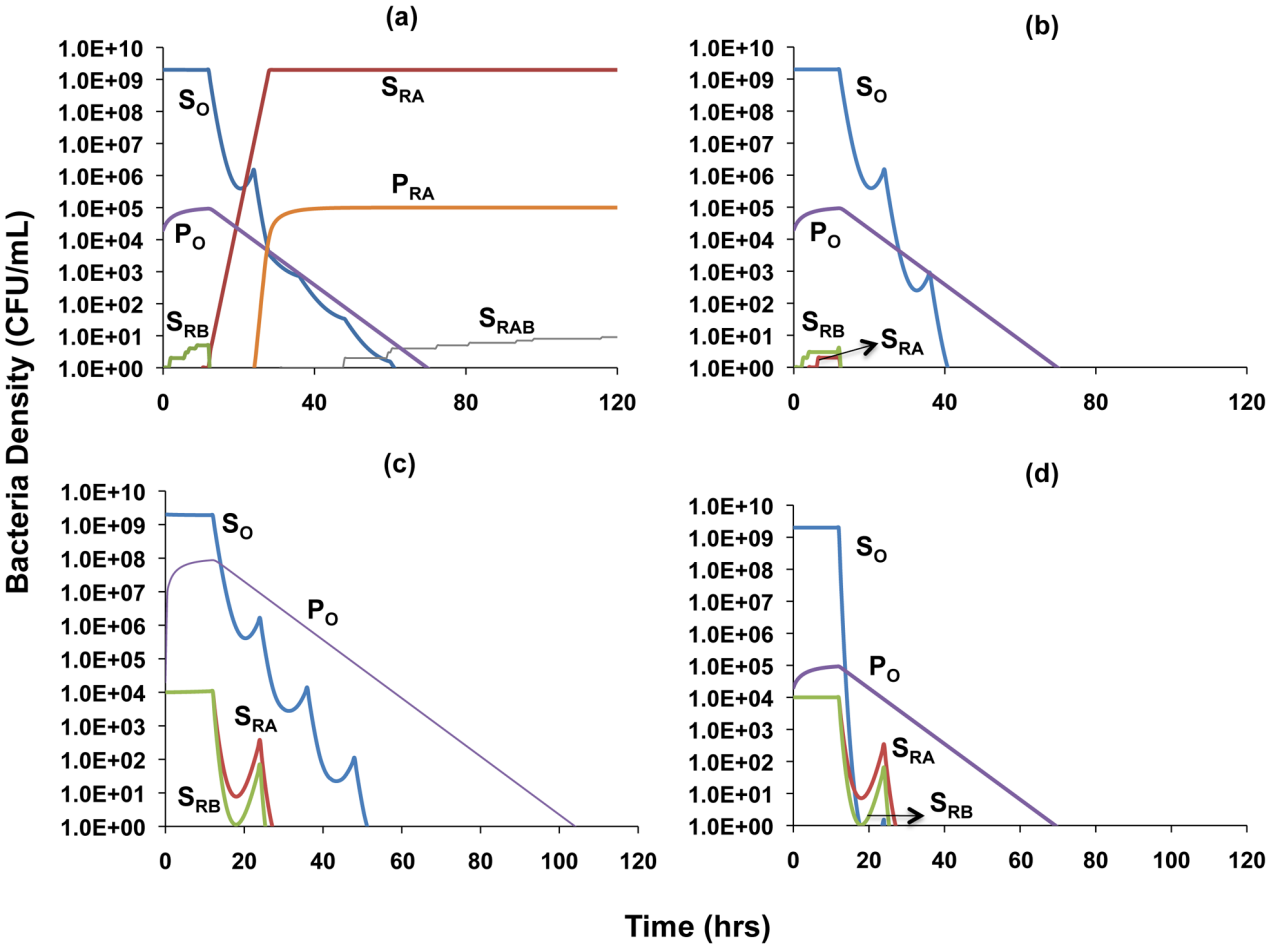
Simulation of the population dynamics of actively replicating and persister bacteria under antibiotic treatment. Unless otherwise noted, parameter values used for the simulations are the standard values in Table 1. (a) Clearance dynamics under single antibiotic treatment, assuming low level persistence (A_max_ = 0, B_max_ = 10, *f* = 10^−5^, *g* = 10^−6^, *ψ_minA_* = 0, *ψ_minB_* = −5) (b) Clearance dynamics under dual antibiotic treatment, assuming additive drug interactions and low level persistence (*f* = 10^−5^, *g* = 10^−6^, *ψ_minA_* = −5, *ψ_minB_* = −5, *ψ_minAB_* = −5) (c) Clearance dynamics under dual antibiotic treatment, assuming suppressive drug interactions and high level persistence (*f* = 10^−2^, *g* = 10^−3^, *ψ_minA_* = −10, *ψ_minB_* = −5, *ψ_minAB_* = −2) (d) Clearance dynamics under dual antibiotic treatment, assuming synergistic interactions and low level persistence (*f* = 10^−5^, *g* = 10^−6^, *ψ_minA_* = −10, *ψ_minB_* = −5, *ψ_minAB_* = −15).

We open this consideration with sample simulations involving single- (Figure 6a) and dual-drug therapy (Figure 6b) to explore the contributions of persistence to the term of therapy and the emergence of resistance. Figure 6a shows that with single-drug therapy, when mutants resistant to the treating drug are present they ascend to high levels and generate concomitant levels of resistant persisters. Since resistance to the second drug is generated by mutation, the large numbers of bacteria resistant to the treating drug can allow for the generation of a minority population of bacteria resistant to both drugs. With two-drug therapy the bacteria resistant to single drugs will be eradicated due to their susceptibility to the other antibiotic (Figure 6b). Populations of these single-drug resistant bacteria do not grow to high enough densities to generate persister populations that can influence the clearance dynamics.

We explore the combined roles of exponential-phase cidal dynamics and persistence with a consideration of two extreme cases: (i) a worst case scenario in which the two antibiotics interact suppressively and also lead to a high level of persistence (Figure 6c) and (ii) the best case scenario of synergistic antibiotics that lead to a low level of persistence (Figure 6d). We differentiate between the types of drug interaction by using different values for the maximal death rate that drug combinations engender. To account for the observation that different combinations of drugs generate different levels of persistence, we modulate the persister generation and loss parameters, *f* and *g*, such that increased efficacy for drug combinations in terms of reducing the level of persistence leads to lower values of these parameters. Values of the conversion parameters are chosen such that densities of persisters are in the range of those we observed in our experimental results. To address the fact that most infections are only treated when the number of bacteria is sufficiently great to cause symptoms, and that resistance can be acquired by mutation or horizontal gene/genetic element transfer from the existing flora, in our simulations we assume that at the onset of treatment there are already minority populations of cells resistant to each antibiotic [45]. We also assume that there is a minority population of persister cells present prior to the initiation of therapy.

As can be seen by comparing Figures 6c and 6d, synergistic interactions between antibiotics and a low level of persistence serve to decrease the time to clearance of the infection. Evidenced by the similarities in the decline slopes of the P populations in Figures 6c and 6d, it is worth noting that the rate of clearance of the persister population with synergistic antibiotics is similar to that with suppressive drugs. However, the synergistic antibiotics are able to eradicate the persister population more rapidly by more efficiently reducing the numbers of the sensitive population that replenishes lost persister cells. Mutants simultaneously resistant to both drugs do not arise because the number of cells in the populations resistant to single drugs and their persisters remain too low to generate doubly resistant mutants.

### The contribution of a spatial refuge

**Figure 7:**
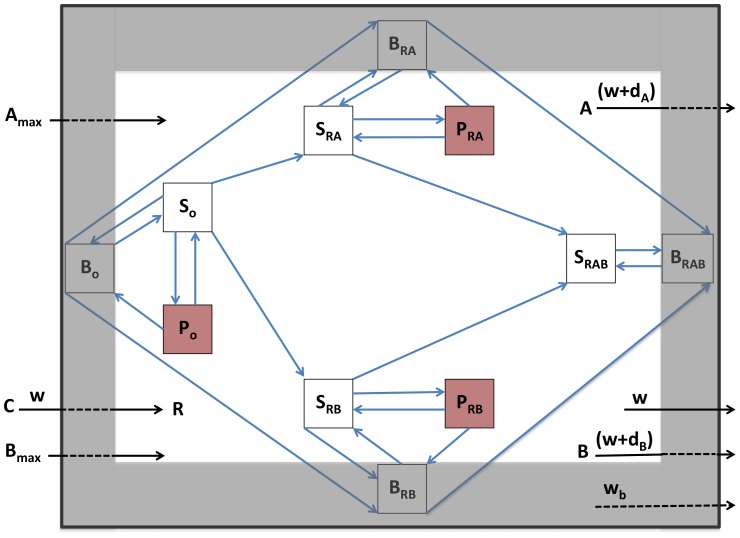
Schematic diagram of the population and evolutionary dynamic model of two-drug therapy. S_x_, actively-growing bacteria; P_x_, persisters; B_x_, bacteria in spatial refuge; x = O, sensitive to both antibiotics; x = RA, resistant to antibiotic A; x = RB, resistant to antibiotic B; x = RAB, resistant to both antibiotics. C, resource reservoir; R, internal concentration of resource; A_max_ and B_max_, concentration of antibiotic periodically added; A and B, internal concentration of antibiotics, d_A_ and d_B_, antibiotic decay rates; w, flow rate, main compartment; w_b_, flow rate, spatial refuge.

The above situation, where the entire population is exposed to the same level of the antibiotic is an idealized one that may be met in flasks, but is unlikely in patients. For many infections, perhaps the majority, antibiotics will not have complete access to the infecting population of bacteria. Some bacteria may be in abscesses, empyema or embedded as non/slowly-dividing cells in biofilms [46], [47]. To account for this, we extend the model to allow for another population of bacteria, B, which occupy a spatial refuge and are thereby less responsive to the antibiotics than the planktonic population. Bacteria in this subpopulation are generated deterministically from both S and P cells at a rate of *f_b_* per hour and return to the actively replicating population at a rate of *g_b_* per hour. We assume that bacterial growth rate is decreased in the refuge and that bacterial susceptibility to antibiotics is proportional to their growth rate [48]. As such, the decrease in maximal growth in the refuge population (*ψ_maxb_*) is paralleled by an equivalent quantitative increase in the MIC of antibiotics in that compartment. Resources enter this refuge and the bacteria within are washed out at rate *w_b_* per hour (*w_b_*<*w*). We show a schematic of this two-compartment model in Figure 7. The complete set of equations is available in Text S1.

**Figure 8:**
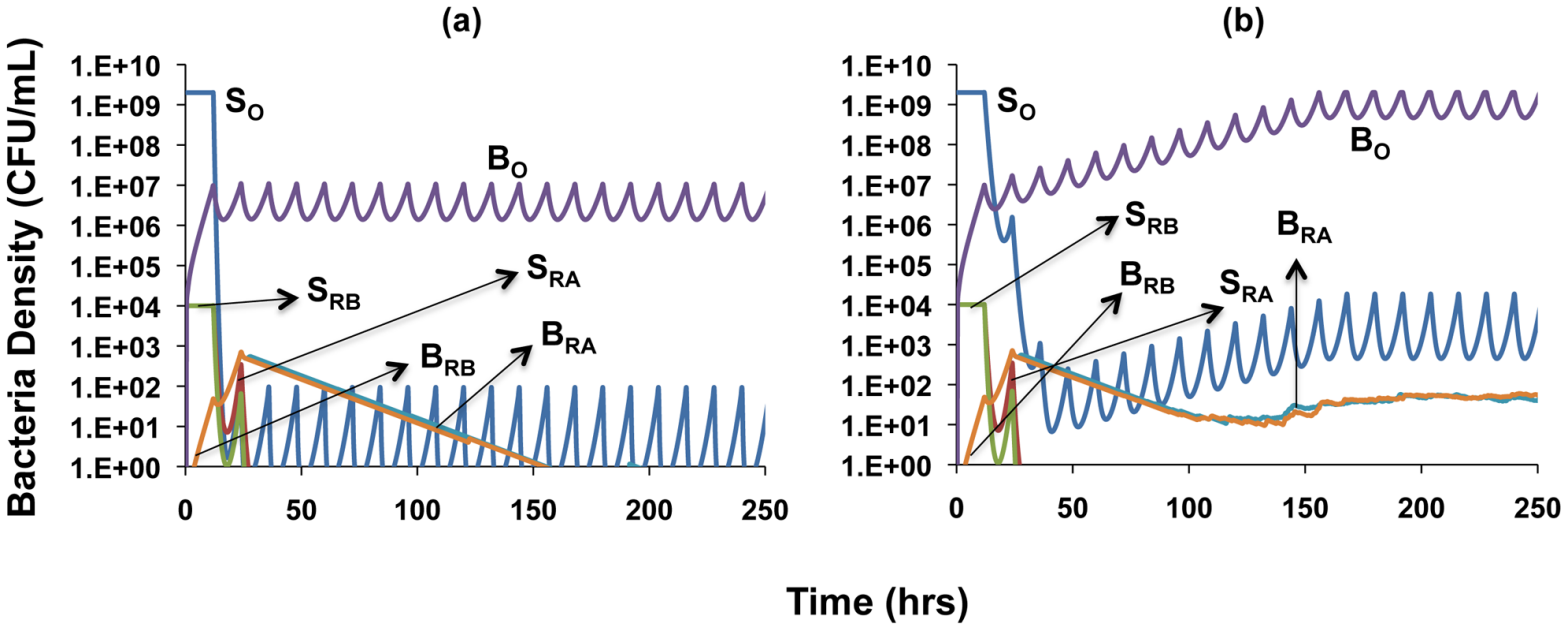
Simulation of the population dynamics of actively replicating and spatial refuge bacteria under antibiotic treatment. Unless otherwise noted, parameter values used for the simulations are the standard values in Table 1. For subpopulations in the spatial refuge, *ψ_maxb_* = 0.5, *w_b_* = 0.05, *f_b_* = 10^−5^, *g_b_* = 10^−6^, *MIC_A_* = 3, *MIC_B_* = 3, *MIC_AB_* = 3. (a) Clearance dynamics under dual antibiotic treatment, assuming synergistic drug interactions *(ψ_minA_* = −10, *ψ_minB_* = −5, *ψ_minAB_* = −15) (b) Clearance dynamics under dual antibiotic treatment, assuming suppressive drug interactions (*ψ_minA_* = −10, *ψ_minB_* = −5, *ψ_minAB_* = −2).

We consider the role of the refuge with simulation runs using the same parameters and initial conditions as in the single compartment simulation, Figures 6c and 6d, but now allow bacteria to migrate to a refuge at the same rates at which persisters are formed. Contrary to the results shown in Figure 6, the infections are not cleared, and susceptible bacteria in both the refuge and the planktonic compartment oscillate around constant densities (Figures 8a and 8b). This result obtains because for both physiological (decreased replication rate) and spatial (reduced antibiotic access) reasons, bacteria in the refuge are more refractory to antibiotics than a more transient planktonic persister subpopulation which continually reverts to a rapidly growing state. It should be noted though, that the infections can be cleared by either increasing antibiotic dose or decreasing the rate of migration of cells into the refuge (Figure S5).

A comparison of Figures 8a and 8b shows an effect of the type of interaction between antibiotics. The susceptible cells are maintained at a lower density when the drug interaction is synergistic (Figure 8a) than when it is suppressive (Figure 8b). Also, while the single-drug resistant mutants are eliminated under synergistic interactions (Figure 8a), they are maintained when the interaction is suppressive (Figure 8b). Under the latter conditions, the population of susceptible cells is maintained at a high enough density to continually generate single-drug resistant mutants. However, since the single-drug resistant bacteria remain susceptible to the activity of the other drug, we do not record any instances of dual-drug resistance in these simulations regardless of whether interactions are synergistic or suppressive.

## Discussion

The rational design of multi-drug antibiotic therapy requires information about the pharmacodynamics of the component drugs individually and in combination as well as how those drugs will affect the population and evolutionary dynamics of the target bacteria. In this study, we use *in vitro* pharmacodynamic experiments with *E. coli* and *S. aureus* to explore the pharmacodynamics of single and pairs of antibiotics of different classes. Using mathematical models and computer simulations, we explore how the observed pharmacodynamics will affect the microbiological course of therapy and evolution of resistance. Here we briefly summarize these theoretical and experimental results and discuss their potential implications for multi-drug therapy.

### Pharmacodynamics

We use Hill functions to characterize the relationship between the concentrations of single and pairs of drugs and the rates of kill of the target bacteria during the initial, exponential, phase of exposure. The concentrations of both single and pairs of drugs are expressed as single variables, multiples of cidal units. These cidal units are, for single drugs, equivalent to multiples of Clinical and Laboratory Standards Institute (CLSI) [49] estimates of their MICs; for pairs of drugs, they are sums of equipotent concentrations of the two drugs (equal multiples of their respective CLSI MICs). This formulation allows a comparison of the cidal/inhibitory activities of drugs in combination with those of their component single drugs at equivalent concentrations. Using this method we characterize and quantify the form of the interaction between pairs of drugs, synergy, antagonism, suppression or additivity.

Our experimental results illustrate the necessity of comprehensive empirical PD assessments for drug combinations rather than attempting to predict their interactions *a priori* or based on single interaction parameters. In experiments with *E. coli*, drug combinations exhibited concentration-dependent synergy, antagonism and suppression in ways that, for most combinations, could not have been predicted from current understanding of the mechanisms of drug action. For example, it is generally assumed and seemingly reasonable to anticipate that when mixed with drugs that are bacteriostatic, like chloramphenicol, antibiotics that require cell division for their action, like the beta lactams, will not be as effective in killing bacteria than when they are alone [50]. Unfortunately, the classification of antibiotics as bactericidal or bacteriostatic is not as clear in practice as is often alluded to [51]. For example, in our *E. coli* experiments, tetracycline, which is often classified as bacteriostatic [26], was clearly bactericidal at higher concentrations, more so than ampicillin, which is a member of the presumptively bactericidal beta-lactam family of drugs. The combination of tetracycline and ampicillin was more effective in killing bacteria than ampicillin alone, albeit less so than tetracycline on its own. On the other hand, combinations of tetracycline with ciprofloxacin or tobramycin were less effective than either of these drugs alone.

For *S. aureus* we only observed antagonistic and suppressive interactions for all six pairs of drugs considered. With two exceptions, the efficacy of the combinations of drugs was intermediate between that of the most and least bactericidal. The exceptions are noteworthy; vancomycin in combination with either ciprofloxacin or oxacillin exhibited suppressive interactions. Most dramatically, the combination of vancomycin and oxacillin had virtually no bactericidal activity. This is a good illustration of the point we made earlier, that based on the PD of these single drugs we could not have predicted how they would interact in combination.

It is clear from single drug studies that the level of persistence depends on the antibiotics and their concentrations [41]. While the present experiments support this interpretation, they are also consistent with the proposition that there is no way to predict how two drugs will interact to determine the level of persistence. What is clear from our results is that the density of persisters with two-drug combinations will be no greater than that of the single drugs alone. For most combinations, the density of persisters was intermediate between that of the two antibiotics or at a level similar to that observed for the component drug that generated a lower level of persistence. This suggests that the component antibiotics determine the lower and upper limits for the density of persisters when drugs are combined. Interestingly, there is limited correlation between the pharmacodynamic efficacy of combinations in the exponential, cidal, phase of the encounter between the bacteria and drugs and the level of persistence. As suggested earlier for the kill phase of the pharmacodynamics, the physiological and molecular reasons for this are unclear.

### Population and evolutionary dynamics and potential implications for treatment

Our mathematical and computer simulation model of the pharmaco-, population and evolutionary dynamics of bacteria undergoing dual drug therapy illustrates how the interactions between drugs affect the microbiological course of treatment. Drug combinations that exhibit suppressive interactions in either the rate of kill and/or level of persistence will require more time to clear an infection than synergistic drugs. From the perspective of treatment, persistence is a refuge from the cidal action of the antibiotics. If that refuge is small, i.e. the persistence level is low, it will have little effect on the rate of clearance. On the other hand, a high level of persistence serves as a substantial refuge that continually re-seeds the treated population and lengthens the term of therapy. Our analysis suggests that in general, while persisters may retard the rate at which bacteria are cleared, they are unlikely to prevent clearance. This, however, should not be interpreted to suggest that persistence cannot lead to treatment failure, since the magnitude of morbidity and the probability of mortality increases with the term of the infection. Lengthier treatment durations can also increase the likelihood of patient non-adherence and thus increase the probability of exposure to sub-therapeutic concentrations of antibiotics. Recent work by two of the authors (PJTJ and BRL) suggests that these sub-MIC concentrations can enrich bacterial populations for existing persisters and also promote the generation of persisters and thereby increase their density in treated populations [41]. Most importantly, there is evidence from clinical studies that supports the proposition that in addition to delaying clearance, persistence may also lead to treatment failure [35], [52]–[54].

In addition to subpopulations of bacteria that are physiologically refractory because they are not growing or growing slowly, there are also subpopulations that, for spatial or other reasons, are less accessible to antibiotics than the dominant population. In our simulations we show that the presence of these refugia can prevent clearance by treatment regimens that lead to clearance in their absence. This has in fact been observed for chronic infections with physically-structured subpopulations of bacteria, such as endocarditis and osteomyelitis, and also for catheter and other foreign-body associated infections [55]. As with persistence, our models indicate that treatment with synergistic combinations of drugs can improve the microbiological course of treatment, i.e. reduce the densities of bacteria in chronic infections relative to suppressive combinations.

A traditional reason for using multiple, rather than single, antibiotics is to prevent the ascent of bacteria resistant to single antibiotics. The results of our simulations support this interpretation of the evolutionary utility of two-drug therapy. Although in our simulations mutants resistant to single drugs were initially present at low frequencies, these cells were either cleared or remained minority populations. Further, with the parameters employed, two-drug resistance never emerged. The reason for the latter is that the populations of single-drug resistant bacteria and their corresponding persister and refuge subpopulations remained in check by the drug to which they were susceptible. They did not grow to high enough numbers to generate multi-drug resistance via mutation. This evolutionary benefit of two-drug therapy obtained even when the drugs suppressed each other's activity. Indeed, there exists some experimental evidence to suggest that antagonistic and suppressive drug combinations may be even more efficient than synergistic combinations in preventing evolution of multi-drug resistance [28]. When interactions are synergistic, evolution of resistance to one of the drugs aborts the enhancing effect of the other, whereas with antagonistic interactions single-drug resistance removes the suppressive effect on the drug to which those mutants are susceptible [28], [56].

Of note though; while in the absence of refugia two-drug therapy can lead to the clearance of minority populations of single-drug resistant bacteria, this need not be the case when there are refugia. As a consequence of these refugia, the number of bacteria sensitive to both antibiotics can remain sufficiently large to continually seed the population with mutants resistant to single drugs. Whether or not this will occur depends on the nature of the two-drug interactions. Suppressive drugs, because they lead to greater densities of susceptible cells, are more likely to allow for the continuous repopulation of single–drug resistance by mutation.

### Caveats and limitations

At best, *in vitro* pharmaco- and population dynamics experiments and mathematical modeling and simulation studies of the sort presented here can only provide a rational and necessarily quantitative base for the design of antibiotic treatment protocols. The within-host model we use here, for instance, does not explicitly consider the contribution of the innate or adaptive immune systems to clearance. Ultimately the evaluation of these protocols has to be made in treated animals where the immune system contributes to the clearance of the infection and, alas, the pathology [57].

The approach we have used in both the experimental and modeling elements of this study have been phenomenological, they do not incorporate or address the physiology and molecular mechanisms of action of single antibiotics or interactions between antibiotics in inhibiting the growth and killing their target bacteria. We justify this approach in two ways: First from the practical perspective of antibiotic treatment, the phenomenology considered, the relationship between the concentrations of single and multiple antibiotics in inhibiting the growth and killing the bacteria is more important than an understanding of the mechanisms responsible. Second, despite all that is known about the targets of antibiotic action and how they are related to the molecular structure of these compounds, we still know relatively little about how antibiotics inhibit the growth of and kill bacteria, see for example [58]. Similarly, in our consideration of persisters we assume that these bacteria are generated stochastically, and do not explicitly account for deterministic mechanisms such as stress responses [59], [60] that can also contribute to persister generation. This approach has the virtue of simplifying the model while still maintaining its quantitative integrity, since the levels of persisters generated in the simulations are equivalent to those observed experimentally.

For convenience and tractability, in our model we treated susceptibility and resistance as discrete states with different pharmacodynamic properties. In reality bacterial susceptibility and resistance to antibiotics is a continuum that depends not only on the specific target of the drug, but also the rates at which cells take up and remove these compounds, e.g. via efflux pumps. In some cases, single mutations in regulatory loci or efflux systems can simultaneously reduce the susceptibility of bacteria to multiple antibiotics [61], [62]. Multi-drug resistance may also be acquired in a single step by the horizontal transfer of genes or accessory genetic elements from the resident flora [63], [64]. Another noteworthy caveat is that for some infections, bacterial population sizes may well exceed the numbers we have considered here, thereby increasing the likelihood that mutants resistant to two antibiotics will be generated. As intriguing as they may be, a formal consideration of these realities is beyond the scope of this study.

## Materials and Methods

### Bacterial strains and growth/sampling media

Experiments involving *E. coli* were conducted using strain 018:K1:H7 (designated CAB1) that was originally isolated from a child with meningitis and supplied by Craig A. Bloch [65]. This strain has been used in previous studies of the within-host pharmacodynamics of antibiotic and phage treatment [27], [44], [66]. The experiments involving *Staphylococcus aureus* were conducted using strain Newman which was isolated from a patient with osteomyelitis and generously provided by Dr. William Shafer. Bacteria were grown in 10 mL of Lysogeny Broth (LB) (*E. coli*) or Mueller-Hinton II (MHII) broth (*S. aureus*) in 50-mL Pyrex flasks at 37°C with aeration and shaking (200 rpm). Viable cell densities in bacterial cultures were determined by plating dilutions (made in 0.85% saline) on LB Agar.

### Antibiotics

For experiments involving *E. coli*, 10 µg/mL stock solutions of ciprofloxacin, ampicillin, tobramycin and tetracycline were diluted in fresh LB to appropriate concentrations for each experiment. Antibiotic stocks used in the *S. aureus* experiments were prepared to a final concentration of 10 µg/ml for ciprofloxacin, gentamicin and oxacillin while vancomycin was prepared to a final stock concentration of 15 µg/ml. Dilutions of requisite antibiotics were made fresh in MHII broth to the appropriate concentrations for each experiment. All antibiotics were procured from Mediatech, Inc. (Herndon, Va.) and Sigma-Aldrich (St. Louis, Mo.).

### MIC determination

Minimum Inhibitory Concentrations (MIC) for *E. coli* CAB1 and *S. aureus* Newman were estimated using the broth microdilution procedure recommended by the Clinical and Laboratory Standards Institute (CLSI) [49].

### Antibiotic time-kill experiments

Overnight cultures of *E. coli* CAB1 were diluted 1∶2000 into fresh LB to initiate exponential growth, and were allowed to grow to a final density of approximately 1×10^7^ cells per mL before antibiotics at desired concentrations were added. For single drug experiments, 0, 0.2×, 0.5×, 1×, 2.5×, 5× and 10 multiples of MIC (xMIC) were added to each culture, and for dual drug time kill experiments, pairs of antibiotics were combined to generate solutions that contained 0, 0.2×, 0.5×, 1×, 2.5×, 5× and 10×MIC of each antibiotic. The cultures were sampled to estimate viable cell densities every 10 min for the first 1 h, every 30 min for the next 2 h, and at 6 h. Overnight *S. aureus* Newman cultures were diluted to a final concentration of ∼1×10^7^ bacteria per ml in fresh MHII media and incubated for 1 hour at 37°C shaking at 200 rpm to ensure entry into the exponential growth phase. Cultures were then inoculated with 0, 0.1×, 0.5×, 1×, 2.5×, 5× and 10×MIC of each antibiotic individually and then in pairs of equal concentrations for the dual treatment. Viable cell densities were estimated every 10 minutes for the first hour and then every 30 minutes for the next 5 hours.

### Level of persistence experiments

In order to assess the level of persistence, we conducted late-term time kill experiments using 10 independent replicate cultures for each drug and drug pairing. Experiments were initiated as described in the aforementioned time-kill assays, but sampling was done at a single time point - 6 h for *E. coli* and 22 h for *S. aureus*. Sampling at these time points has been previously shown to provide good estimates for persisters in a culture [41], [67]. We also confirmed that, with the protocol used, there were no drug carryover effects on plating efficiency.

### Pharmacodynamic functions

As in Regoes *et al.* [27], we use a four-parameter Hill function-based pharmacodynamic function (Equation 1) to characterize the exponential phase death rate engendered by the antibiotic(s) singly and in pairs,
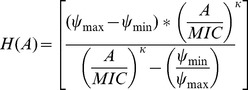
(1)where *ψ_max_* is the maximum bacterial growth rate in the absence of antibiotics, *ψ_min_* is the maximum death rate generated by the antibiotic, *κ* describes the sigmoidicity of the Hill function, the *MIC* is the pharmacodynamic minimum inhibitory antibiotic concentration, and *A* is the antibiotic concentration. In this study, the concentrations of single antibiotics are presented as multiples of the MICs as estimated by standard CLSI serial dilution procedures. For pairs of drugs, A is equal to the sum of equal multiples of the component single drug CLSI estimated MICs. For both single and two drugs, we use exponential phase time kill data for different multiples of the CLSI MICs and the procedure in [27] to generate Hill functions and estimate their parameters. Thus for each single drug, we have two estimates of MIC, that obtained by serial dilution and the realized MIC (rMIC) estimated from the Hill function. For pairs of drugs we only have single estimate of the minimum inhibitory concentration, that obtained by fitting the Hill function, rMICs.

For single drugs and for drug pairs, net bacterial growth rates under antibiotic action are described by the following respective equations:
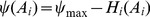
(2)

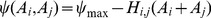
(3)


## Supporting Information

Figure S1 **Time-kill curves of** ***E. coli*** **CAB1 exposed to single antibiotics.** Changes in viable cell density for cultures treated with varying concentrations (0.2×CU, 0.5×CU, 1×CU, 2×CU, 5×CU and 10×CU). Each multiple of cidal unit (xCU) is equivalent to the corresponding multiple of MIC (xMIC). (a) ampicillin (b) ciprofloxacin (c) tetracycline (d) tobramycin.
(TIF)

Figure S2 **Time-kill curves of** ***E. coli*** **CAB1 exposed to pairs of antibiotics.** Changes in viable cell density for cultures treated with varying concentrations (0.4×CU, 1×CU, 2×CU, 5×CU, 10×CU and 20×CU) of each antibiotic pair. Each multiple of cidal unit (xCU) is equivalent to the sum of equal multiples of MIC (xMIC) of each drug, e.g. 1×CU is the combination of 0.5×MIC of each antibiotic. (a) ampicillin+ciprofloxacin (b) ampicillin+tetracycline (c) ciprofloxacin+tetracycline (d) ciprofloxacin+tobramycin (e) ampicillin+tobramycin (f) tetracycline+tobramycin.
(TIF)

Figure S3 **Time-kill curves of** ***S. aureus*** **Newman exposed to single antibiotics.** Changes in viable cell density for cultures treated with varying concentrations (0.1×CU, 0.5×CU, 1×CU, 2×CU, 5×CU and 10×CU) of each antibiotic. Each multiple of cidal unit (xCU) is equivalent to the corresponding multiple of MIC (xMIC). (a) ciprofloxacin (b) gentamicin (c) oxacillin (d) vancomycin.
(TIF)

Figure S4 **Time-kill curves of** ***S. aureus*** **Newman exposed to pairs of antibiotics.** Changes in viable cell density for cultures treated with varying concentrations (0.2×CU, 1×CU, 2×CU, 5×CU, 10×CU and 20×CU) of each antibiotic pair. Each multiple of cidal unit (xCU) is equivalent to the sum of equal multiples of MIC (xMIC) of each drug, e.g. 1×CU is the combination of 0.5×MIC of each antibiotic. (a) gentamicin+ciprofloxacin (b) ciprofloxacin+oxacillin (c) ciprofloxacin+vancomycin (d) gentamicin+oxacillin (e) gentamicin+vancomycin (f) oxacillin+vancomycin.
(TIF)

Figure S5 **Effects of increasing dose and decreasing rates of migration into spatial refuge on clearance dynamics.** Unless otherwise noted, parameter values are the same as those used for corresponding simulations shown in Figure 5. (a) Clearance dynamics with a higher dose of antibiotics, assuming synergistic interactions (Amax = 10, Bmax = 10) (b) Clearance dynamics with a higher dose of antibiotics, assuming suppressive interactions (A_max_ = 10, B_max_ = 10 (c) Clearance dynamics with a lower rate of migration of cells into the spatial refuge assuming synergistic interactions (*f_b_* = 10^−6^, *g_b_* = 10^−7^) (d) Clearance dynamics with a lower rate of migration of cells into the spatial refuge assuming suppressive interactions (*f_b_* = 10^−6^, *g_b_* = 10^−7^)
(TIF)

Table S1 Pharmacodynamic function parameter estimates and standard errors for *E. coli* experiments.
(DOC)

Table S2 Pharmacodynamic function parameter estimates and standard errors for *S. aureus* experiments.
(DOC)

Text S1 Differential equations used for simulation of the two-compartment mathematical model.
(DOCX)
