# Supplementary figures and images for: The Pharmaco –, Population and Evolutionary Dynamics of Multi-drug Therapy: Experiments with S. aureus and E. coli and Computer Simulations
Source: PLoS Pathog. 2013 Apr 4;9(4):e1003300. doi: 10.1371/journal.ppat.1003300 (PMC3617031; doi:10.1371/journal.ppat.1003300)

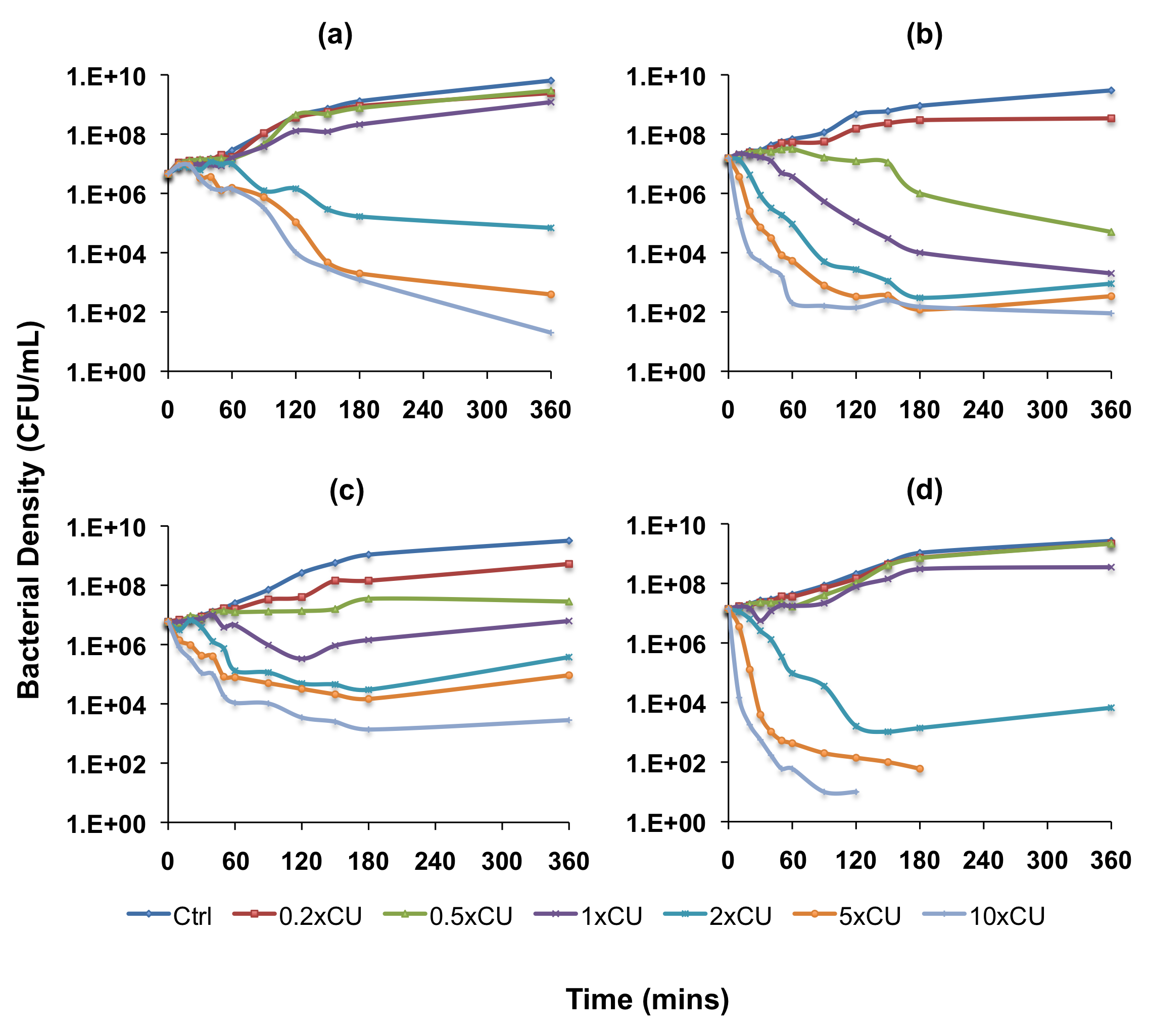

Supplement: Figure S1 — Time-kill curves of E. coli CAB1 exposed to single antibiotics. Changes in viable cell density for cultures treated with varying concentrations (0.2×CU, 0.5×CU, 1×CU, 2×CU, 5×CU and 10×CU). Each multiple of cidal unit (xCU) is equivalent to the corresponding multiple of MIC (xMIC). (a) ampicillin (b) ciprofloxacin (c) tetracycline (d) tobramycin. (TIF) [file ppat.1003300.s001.tif]

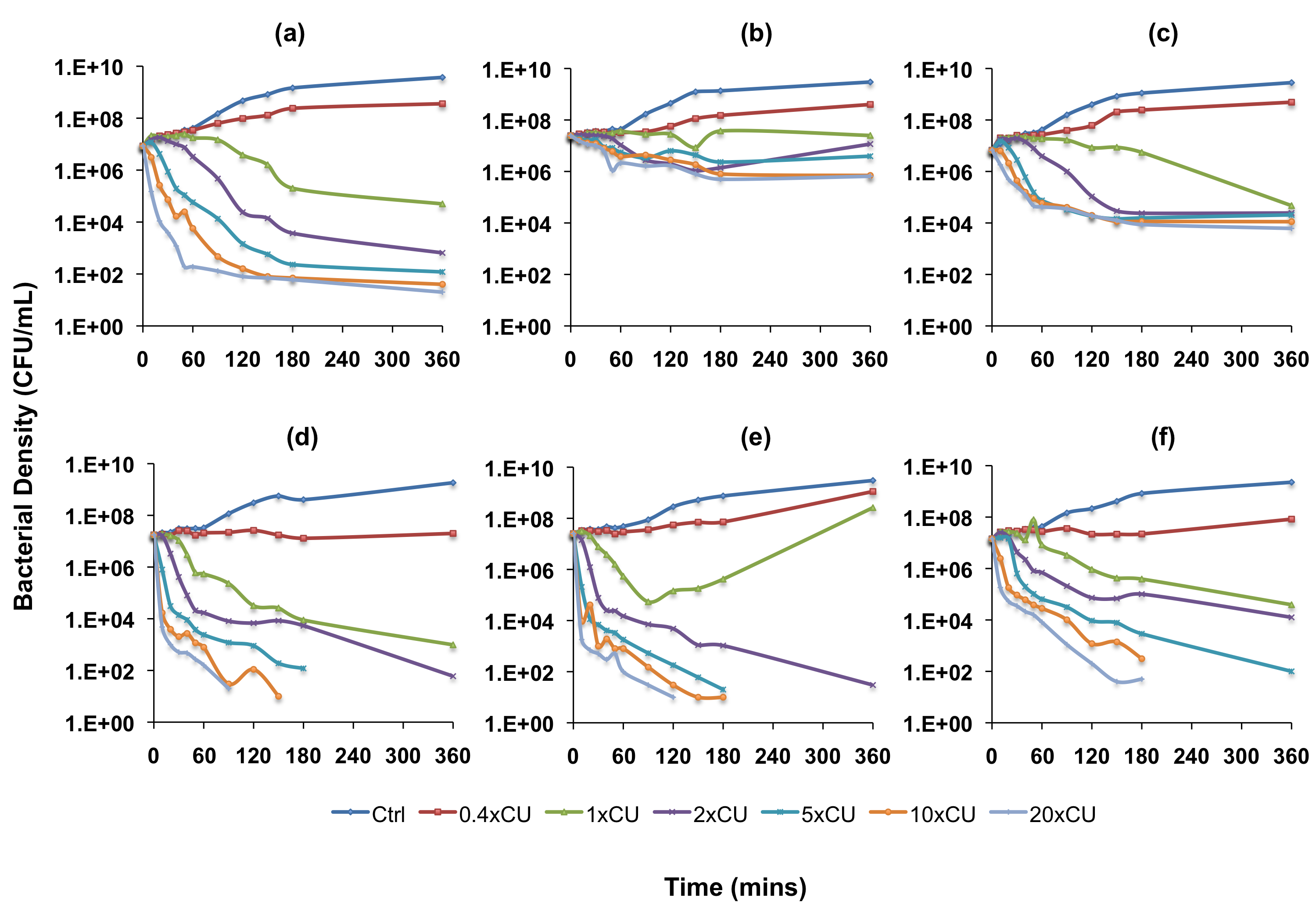

Supplement: Figure S2 — Time-kill curves of E. coli CAB1 exposed to pairs of antibiotics. Changes in viable cell density for cultures treated with varying concentrations (0.4×CU, 1×CU, 2×CU, 5×CU, 10×CU and 20×CU) of each antibiotic pair. Each multiple of cidal unit (xCU) is equivalent to the sum of equal multiples of MIC (xMIC) of each drug, e.g. 1×CU is the combination of 0.5×MIC of each antibiotic. (a) ampicillin+ciprofloxacin (b) ampicillin+tetracycline (c) ciprofloxacin+tetracycline (d) ciprofloxacin+tobramycin (e) ampicillin+tobramycin (f) tetracycline+tobramycin. (TIF) [file ppat.1003300.s002.tif]

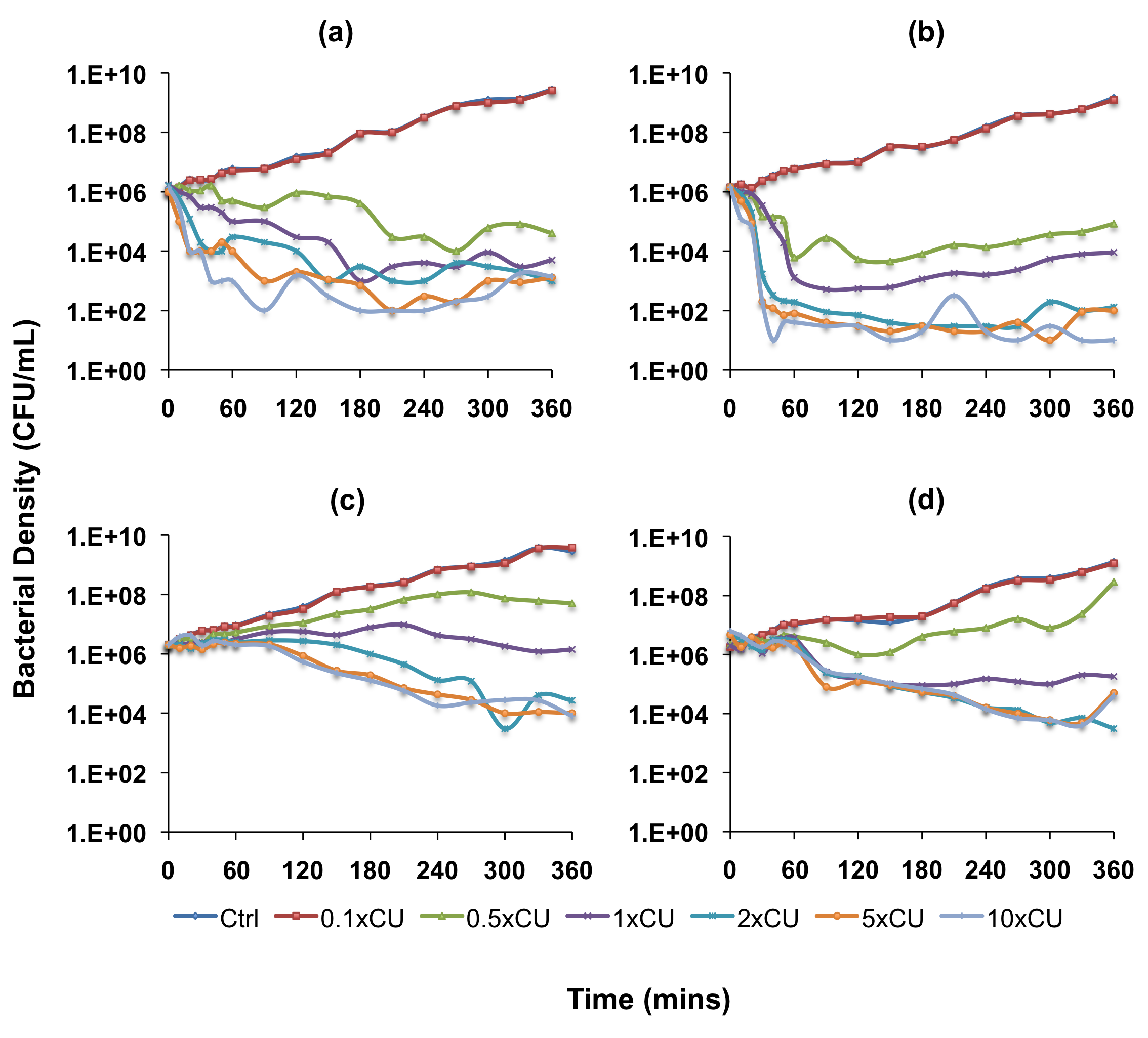

Supplement: Figure S3 — Time-kill curves of S. aureus Newman exposed to single antibiotics. Changes in viable cell density for cultures treated with varying concentrations (0.1×CU, 0.5×CU, 1×CU, 2×CU, 5×CU and 10×CU) of each antibiotic. Each multiple of cidal unit (xCU) is equivalent to the corresponding multiple of MIC (xMIC). (a) ciprofloxacin (b) gentamicin (c) oxacillin (d) vancomycin. (TIF) [file ppat.1003300.s003.tif]

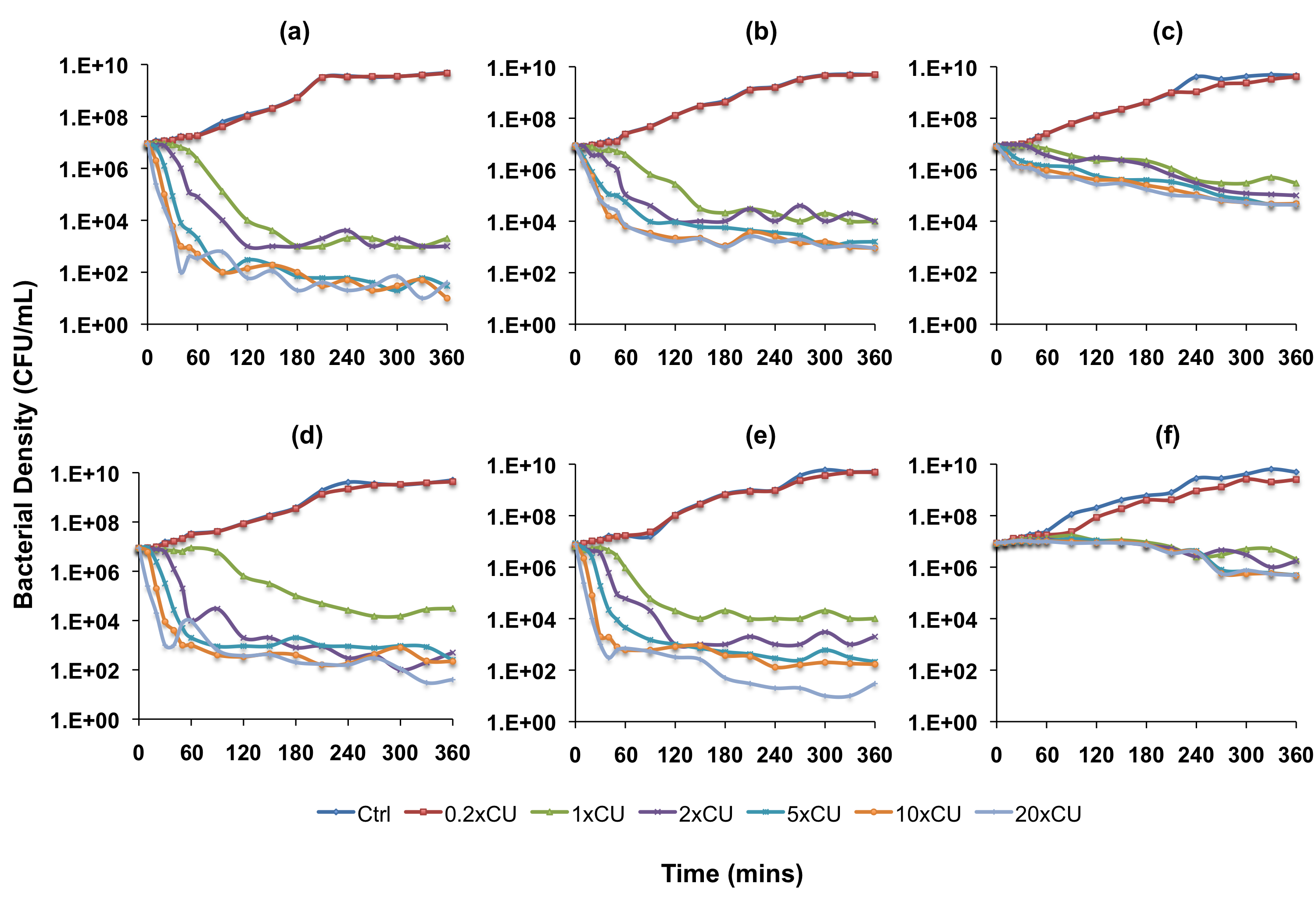

Supplement: Figure S4 — Time-kill curves of S. aureus Newman exposed to pairs of antibiotics. Changes in viable cell density for cultures treated with varying concentrations (0.2×CU, 1×CU, 2×CU, 5×CU, 10×CU and 20×CU) of each antibiotic pair. Each multiple of cidal unit (xCU) is equivalent to the sum of equal multiples of MIC (xMIC) of each drug, e.g. 1×CU is the combination of 0.5×MIC of each antibiotic. (a) gentamicin+ciprofloxacin (b) ciprofloxacin+oxacillin (c) ciprofloxacin+vancomycin (d) gentamicin+oxacillin (e) gentamicin+vancomycin (f) oxacillin+vancomycin. (TIF) [file ppat.1003300.s004.tif]

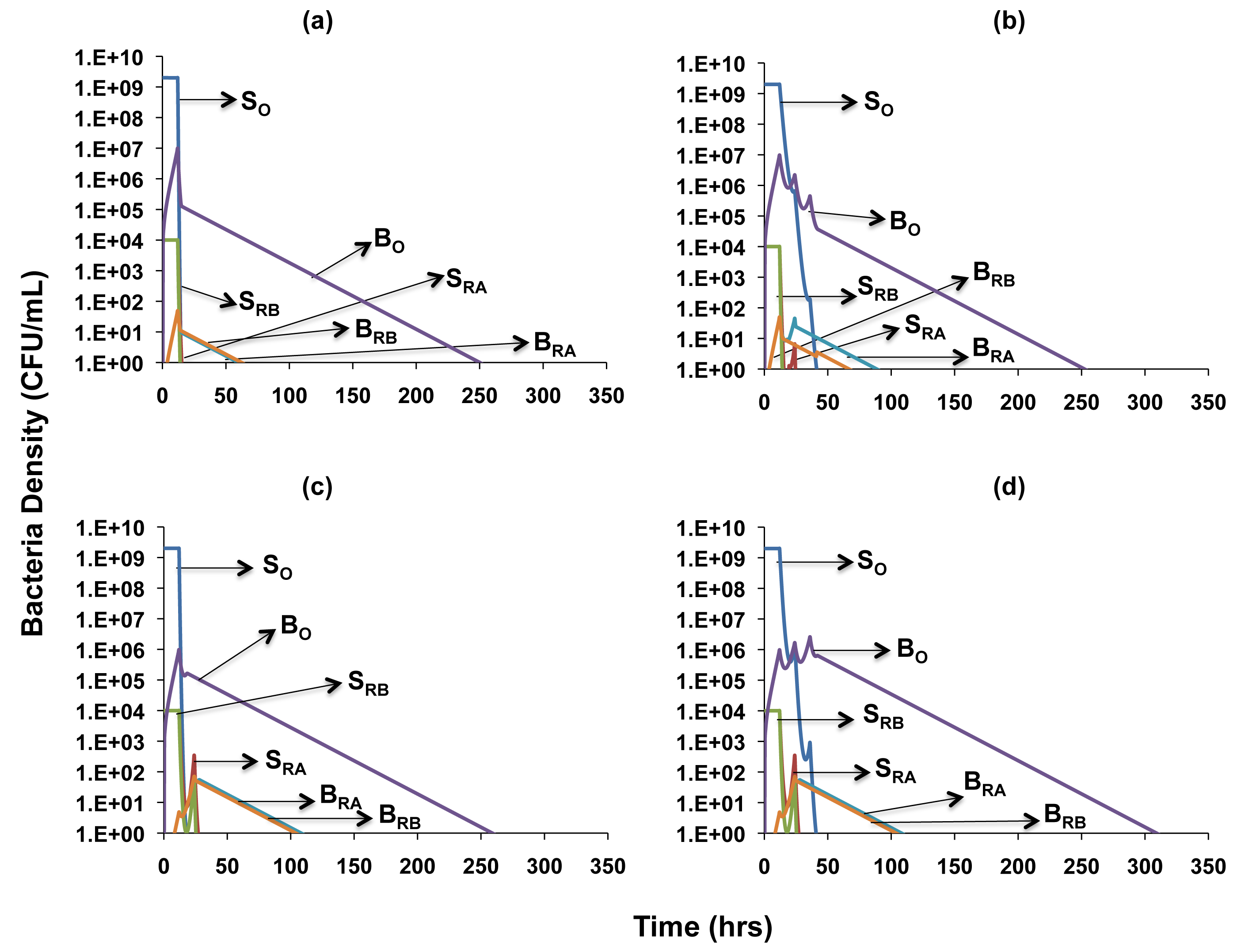

Supplement: Figure S5 — Effects of increasing dose and decreasing rates of migration into spatial refuge on clearance dynamics. Unless otherwise noted, parameter values are the same as those used for corresponding simulations shown in Figure 5. (a) Clearance dynamics with a higher dose of antibiotics, assuming synergistic interactions (Amax = 10, Bmax = 10) (b) Clearance dynamics with a higher dose of antibiotics, assuming suppressive interactions (Amax = 10, Bmax = 10 (c) Clearance dynamics with a lower rate of migration of cells into the spatial refuge assuming synergistic interactions (fb = 10−6, gb = 10−7) (d) Clearance dynamics with a lower rate of migration of cells into the spatial refuge assuming suppressive interactions (fb = 10−6, gb = 10−7) (TIF) [file ppat.1003300.s005.tif]
