# Supplementary material for: The Pharmaco –, Population and Evolutionary Dynamics of Multi-drug Therapy: Experiments with S. aureus and E. coli and Computer Simulations
Source: PLoS Pathog. 2013 Apr 4;9(4):e1003300. doi: 10.1371/journal.ppat.1003300 (PMC3617031; doi:10.1371/journal.ppat.1003300)
Supplement: Table S1 — Pharmacodynamic function parameter estimates and standard errors for E. coli experiments. (DOC) [file ppat.1003300.s006.doc]

Table S1. Pharmacodynamic function parameter estimates and standard errors for *E. coli* experiments.

| Antibiotic(s) | ψ_max_ (h^-1^) | ψ_min_ (h^-1^) | κ | MIC or rMIC |
| --- | --- | --- | --- | --- |
| Ciprofloxacin | 1.59±0.20 | -15.7±1.06 | 1.42±0.14 | 0.70±0.06 |
| Ampicillin | 1.57±0.15 | -1.16±0.30 | 4.53±1.99 | 3.47±0.45 |
| Tetracycline | 1.30±0.15 | -8.32±0.76 | 1.46±0.19 | 0.92±0.08 |
| Tobramycin | 1.08±0.35 | -16.6±1.08 | 2.67±0.48 | 1.20±0.19 |
| Ciprofloxacin + Ampicillin | 1.98±0.48 | -18.0±4.21 | 1.29±0.30 | 1.68±0.30 |
| Ciprofloxacin + Tetracycline | 1.91±0.15 | -5.69±0.22 | 2.56±0.29 | 2.22±0.13 |
| Ciprofloxacin + Tobramycin | 1.43±0.69 | -15.2±1.12 | 1.30±0.25 | 0.34±0.10 |
| Ampicillin + Tetracycline | 1.24±0.23 | -5.96±4.72 | 0.61±0.24 | 1.15±0.25 |
| Ampicillin + Tobramycin | 1.41±0.85 | -15.1±0.99 | 1.58±0.37 | 0.38±0.12 |
| Tetracycline + Tobramycin | 1.48±0.55 | -9.68±1.17 | 1.45±0.38 | 0.92±0.21 |
