# Supplementary material for: The Pharmaco –, Population and Evolutionary Dynamics of Multi-drug Therapy: Experiments with S. aureus and E. coli and Computer Simulations
Source: PLoS Pathog. 2013 Apr 4;9(4):e1003300. doi: 10.1371/journal.ppat.1003300 (PMC3617031; doi:10.1371/journal.ppat.1003300)
Supplement: Table S2 — Pharmacodynamic function parameter estimates and standard errors for S. aureus experiments. (DOC) [file ppat.1003300.s007.doc]

Table S2. Pharmacodynamic function parameter estimates and standard errors for *S. aureus* experiments.

| Antibiotic(s) | ψ_max_ (h^-1^) | ψ_min_ (h^-1^) | κ | MIC or rMIC |
| --- | --- | --- | --- | --- |
| Ciprofloxacin | 1.53±0.27 | -3.01±0.26 | 1.55±0.48 | 0.34±0.07 |
| Gentamicin | 1.28±0.26 | -4.06±0.23 | 1.62±0.41 | 0.23±0.05 |
| Oxacillin | 1.51±0.04 | -1.04±0.08 | 1.42±0.14 | 1.56±0.08 |
| Vancomycin | 1.07±0.02 | -1.53±0.01 | 2.43±0.08 | 0.56±0.007 |
| Ciprofloxacin + Gentamicin | 1.66±0.47 | -4.26±0.33 | 1.74±0.63 | 0.42±0.13 |
| Ciprofloxacin + Oxacillin | 1.50±0.27 | -2.88±0.18 | 2.05±0.65 | 0.49±0.11 |
| Ciprofloxacin + Vancomycin | 1.46±0.21 | -1.23±0.14 | 1.82±0.59 | 0.70±0.13 |
| Gentamicin + Oxacillin | 1.41±0.19 | -3.67±0.15 | 2.33±0.61 | 0.62±0.10 |
| Gentamicin + Vancomycin | 1.50±0.34 | -3.85±0.25 | 1.86±0.62 | 0.47±0.12 |
| Oxacillin + Vancomycin | 1.41±0.03 | -0.095±0.02 | 3.00±0.50 | 0.81±0.17 |
