# Supplementary material for: The Pharmaco –, Population and Evolutionary Dynamics of Multi-drug Therapy: Experiments with S. aureus and E. coli and Computer Simulations
Source: PLoS Pathog. 2013 Apr 4;9(4):e1003300. doi: 10.1371/journal.ppat.1003300 (PMC3617031; doi:10.1371/journal.ppat.1003300)
Supplement: Text S1 — Differential equations used for simulation of the two-compartment mathematical model. (DOCX) [file ppat.1003300.s008.docx]

Text S1. Differential equations used for simulation of the two-compartment mathematical model.

$$\begin{matrix} \frac{dS_{O}}{dt}=S_{O}(gr_{AB}-w-f_{b}-f-mu_{SA}-mu_{SB})+gP_{O}+g_{b}B_{O} \\ \frac{dS_{RA}}{dt}=S_{RA}(gr_{B}-w-f_{b}-f-mu_{SB})+gP_{RA}+g_{b}B_{RA}+mu_{SA}S_{O} \\ \frac{dS_{RB}}{dt}=S_{RB}(gr_{A}-w-f_{b}-f-mu_{SA})+gP_{RB}+g_{b}B_{RB}+mu_{SB}S_{O} \\ \frac{dS_{RAB}}{dt}=S_{RAB}(\psi-w-f_{b})+g_{b}B_{RAB}+mu_{SB}S_{RA}+mu_{SA}S_{RB} \\ \frac{dP_{O}}{dt}=P_{O}(-w-f_{b}-g)+fS_{O} \\ \frac{dP_{RA}}{dt}=P_{RA}(-w-f_{b}-g)+fS_{RA} \\ \frac{dP_{RB}}{dt}=P_{RB}(-w-f_{b}-g)+fS_{RB} \\ \frac{dB_{O}}{dt}=B_{O}(bgr_{AB}-w_{b}-g_{b}-mu_{BA}-mu_{BB})+f_{b}(P_{O}+S_{O}) \\ \frac{dB_{RA}}{dt}=B_{RA}(bgr_{B}-w_{b}-g_{b}-mu_{BB})+f_{b}(P_{RA}+S_{RA})+mu_{BA}B_{O} \\ \frac{dB_{RB}}{dt}=B_{RB}(bgr_{A}-w_{b}-g_{b}-mu_{BA})+f_{b}(P_{RB}+S_{RB})+mu_{BB}B_{O} \\ \frac{dB_{RAB}}{dt}=B_{RAB}(\psi_{b}-w_{b}-g_{b})+f_{b}S_{RAB}+mu_{BA}B_{RB}+mu_{BB}B_{RA} \end{matrix}$$

Growth Rates:

Mutation Rates:

$$\begin{matrix} mu_{SA}=\mu_{A}\left(\frac{\psi}{\psi_{\max}}\right) \\ mu_{SB}=\mu_{B}\left(\frac{\psi}{\psi_{\max}}\right) \\ mu_{BA}=\mu_{A}\left(\frac{\psi_{b}}{\psi_{\max}}\right) \\ mu_{BB}=\mu_{B}\left(\frac{\psi_{b}}{\psi_{\max}}\right) \end{matrix}$$

Resources:

$$\begin{matrix} \frac{dR}{dt}=w(C-R)-e(gr_{AB}S_{O}+gr_{A}S_{RB}+gr_{B}S_{RA}+\psi S_{RAB}) \\ \frac{dR_{f}}{dt}=w_{b}(C-R_{f})-e(bgr_{AB}B_{O}+bgr_{A}B_{RB}+bgr_{B}B_{RA}+\psi_{b}B_{RAB}) \end{matrix}$$

Antibiotics:

$$\begin{matrix} \frac{dA}{dt}=A(-w-d_{A}) \\ \frac{dB}{dt}=B(-w-d_{B}) \end{matrix}$$
